# Size-independent, between-individual variability in feed ingestion rate in European seabass (*Dicentrarchus labrax)*

**DOI:** 10.1371/journal.pone.0347113

**Published:** 2026-04-16

**Authors:** Joaquim Tomàs-Ferrer, Irene Moro-Martínez, Enrique Massutí-Pascual, Amàlia Grau, Miquel Palmer

**Affiliations:** 1 LIMIA-IRFAP, CAIB, Unitat Associada al CSIC per l’IMEDEA. Av. Enginyer Gabriel Roca, Port d’Andratx (Mallorca), Balearic Islands, Spain; 2 Institut Mediterrani d’Estudis Avançats (IMEDEA), UIB-CSIC. Esporles, Balearic Islands, Spain; 3 Institut d’Investigacions Agroambientals i d’Economia de l’Aigua (INAGEA), UIB-INIA-CAIB. Palma, Balearic Islands, Spain; Universiti Malaysia Kelantan, MALAYSIA

## Abstract

Understanding between-individual variability in energy acquisition is essential for elucidating many ecological processes in wild fish populations and for enhancing the efficiency of aquaculture production. This study explores whether individual variations in feed ingestion rates among group-reared fish can arise from intrinsic fish-specific, size-independent factors. Specifically, we quantify the residual variability in ingestion rate (i.e., the variability beyond body size effects and extrinsic influences) to assess the role of context-independent, stable, intrinsic behavioural differences that may lead to feeding hierarchies. We monitored the individual feeding behaviour of 48 European seabass adult females (*Dicentrarchus labrax)* externally tagged reared in sea cages (6 cages housing 8 fish each) under three feeding levels (two cages per level) over four months. Across 8 repeated feeding trials per cage, fish were offered feed pellets one at a time using an automated feeder, and their individual pellet consumption were video recorded. Using a Bayesian statistical model, we evaluated the fish-specific probability of pellet consumption as a function of body size, temperature, anthropogenic stress and feeding level, while accounting for variation across individuals and feeding trials. Our results showed: i) a substantial and consistent between-individual variability in ingestion rates across feeding trials, and ii) a relevant negative effect of anthropogenic stress on feeding activity. Notably, individual-specific effects, independent of body size and external variables, accounted for over 70% of the variance in ingestion rate, suggesting that intrinsic and stable behavioural differences, indicative of fish behavioural types, may play a central role in shaping feeding hierarchies.

## Introduction

No two fish are alike. Between-individual variability plays a crucial role in population adaptation and persistence [1,2]. Neglecting individual heterogeneity in favour of population-level means can blur key ecological processes and can underestimate the role of individual responses to environmental changes, as well as their contributions to ecosystem functioning [3]. Indeed, accurate forecasting of population dynamics should ideally include a robust assessment of between-individual variability [4–6]. Ecological relevance aside, between-individual variability also bears practical implications for aquaculture, where phenotypic differences among fish often lead to divergent growth trajectories even within the same rearing cage [7]. Such disparities can ultimately reduce production efficiency and profitability [8].

Fish can differ in many biological traits [9,10], including differences in how energy is acquired, managed and invested in different biological processes [11]. In particular, differences in feed ingestion rate will influence the energy available to invest into growth, somatic maintenance, reproduction and behavioural activities [12–14]. Therefore, unsurprisingly, between-individual variation in feed consumption has been widely reported [8,15–18].

Fish feeding behaviour seems to result from the combination of intrinsic and extrinsic factors [14]. Body size is among the most frequently reported factor affecting feed intake and feed intake rate. Larger fish tend to display disproportionate larger consumption shares of the available feed [14,15,19–22]. This pattern has been related with enhanced competitive ability and metabolic demands [3,6,23]. However, the relationship between body size and feed ingestion rate is often blurred [24,25], suggesting that additional factors beyond size itself may modulate feeding behaviour.

Temperature is likely reported as a major environmental factor affecting fish feed ingestion rates [22,26,27], through its effects on many key aspects of metabolism [28–30], which in turn seems to outcome on fish behaviour [31]. Fish feed ingestion rate typically describes a bell-shaped pattern peaking at an optimal temperature [7,32].

Stress is also a well-documented driver that can alter fish feeding behaviour. Both acute and chronic stressors can lead to subsequent appetite suppression; and decreasing fish ingestion rates [14,33–38]. Stressors affecting feeding behaviour can be anthropogenic, notably machine-generated noise [39,40] and chemical pollutants [41]; or social [14], the latter ones occurring, for example, among conspecifics in aquaculture environments by competition, particularly in feed-limited circumstances [42,43].

The existence of intraspecific feeding variability has long been acknowledged in fish populations [44]. However, between-fish variability in feed ingestion rates arising from intrinsic fish-specificities beyond the influences of body size and external drivers has been rarely quantified, in spite that the existence of stable, context-independent differences in feeding behaviour may lead among-fish hierarchy [45]. This gap is probably related with the methodological challenges for assessing fish feed intake at individual level. Reliability of the methodological approaches proposed for wild fish are under debate [46] and the methods developed for captive fish have advantages, limitations and specific applicability. The simplest approach involves rearing fish individually, and allows precise measurement of feed intake, but at the price of neglecting social interactions [19,47–50]. Another approach is the use of radio-opaque markers incorporated into feed pellets to visualize ingested feed via X-radiography, which allows relatively low-invasive quantification of feed intake in group-reared individuals [15–18,51–55]. The third approach is based on video identification of individual fish, which is currently limited to small groups but allows non-invasive and continuous monitoring of the intake and feeding behaviour [20,56–58].

This study aimed to determine whether fish differ consistently in their feed ingestion rates beyond what can be explained by body size or external conditions. To address this question, we conducted eight repeated feeding trials over four months, video-monitoring groups of reared fish to track individual feeding behaviour. By quantifying the residual variability in ingestion rate (i.e., the variability beyond body size and potential external influences), we sought to reveal intrinsic, fish-specific, and temporally stable differences in feeding behaviour. This approach provides a direct assessment of whether individual fish exhibit consistent behavioural traits related to feed ingestion.

## Materials & methods

### Fish husbandry

This study takes the European seabass (*Dicentrarchus labrax*) as a model species. It is an eurythermal marine fish that lives in shallow waters from the North-Eastern Atlantic to the Mediterranean and the Black Sea. This species is of high commercial interest both for fisheries and aquaculture, the latter accounting for 96% of the total production [59], with farming widely spread throughout the Mediterranean area [60], where is one of the most reared fish since the 1980’s [61].

**Fig 1:**
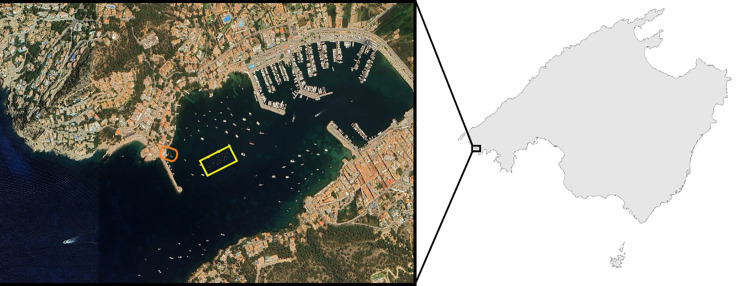
Location of the experimental sea cages (yellow rectangle) and IRFAP-LIMIA facilities (orange oval) within the Port d’Andratx marina and location of Port d’Andratx within the island of Mallorca (Balearic Islands). The navigation channel within the marina can be seen occupying the central axis of the harbour.

All the animals monitored were supplied by an aquaculture company (Aquicultura Balear S.A.U.) located at Palma (Balearic Islands), reared in inland facilities. They were transported as adults to the IRFAP-LIMIA facilities at Port d’Andratx (Balearic Islands) in February, 6 months prior to the start of the experiment. A total of 48 all-female seabass specimens originating from the same family batch were used, ensuring a common genetic background among individuals. They were reared in 6 sea cages (8 m^3^ each), located 200 m from the shoreline, within the inner waters of a marina (Fig 1), where the mean water temperature fluctuates annually between 11.0 and 29.8 ºC. At the beginning of the experiment, fish were 4 years and 1 month old, had a mean body weight of 2,486 ± 376 g and a mean total length of 57.3 ± 3.2 cm.

### Experimental design

Fish were randomly distributed into 6 groups of 8 individuals each. Each fish was tagged with an external 6 mm long polyolefin T-bar spaghetti-tag with a unique colour code (*FD-94, Floy Tag, USA*). Tags were applied with a needle tagging gun (*Mark II Long Pistol, Floy Tag, USA*), inserted below the second dorsal fin, with prior tranquilisation of fish with a phenoxyethanol dose of 600 mg/L for 2 min. Although biofouling developed on tags over time, it was removed monthly with a sponge, also with prior tranquilisation with a phenoxyethanol dose of 600 mg/L for 2 min. Occasional tag loss was anticipated by additionally implanting all fish with a subcutaneous passive integrated transponder (PIT) tag (*ID-100A Microtransponder, Trovan*) as a backup identification method (only 2 tags fell off during the acclimation and trials period). These groups were established 3 months before monitoring to enable the establishment of stable social hierarchies, as previous studies have reported the presence of feeding hierarchies after two months of monitoring in grouped fish [62]. Therefore, the formation of stable feeding hierarchies was assumed at the onset of the monitoring. Feeding trials were conducted from early August to early November, covering a temperature range from 21.8 to 27.6 ºC.

Fish were fed a commercial seabass broodstock dry pellets diet (*Vitalis Repro, Skretting*). Pellets had a mean weight of 0.71 ± 0.02 g, a digestible energy of 19.1 KJ/g, and a 9% water content. Daily rations were adjusted for biomass and temperature, following the manufacturer guidelines, which oscillated between 0.59% and 1.14% of the cage biomass, depending on temperature. Fish were hand-fed 6 days a week in a single daily meal with varying proportions (diet levels (*D*): 60%, 75%, and 90%, 2 cages each) of the recommended daily ration, in order to promote some degree of between-fish competition.

For the feeding trials, a custom-built automatic feeder containing a screw conveyor system and equipped with an underwater camera was used. The feeder was left floating at the centre of the sea cage during the trial (fixed by two crossing ropes), and delivered feed pellets one at a time at a rate of 4 pellets/min, allowing enough time for a single pellet to sink and either being consumed by one of the fish or pass through the net bottom, the latter taking about 10–12 seconds, so there was not more than 1 pellet at a time in the sea cage. The camera was positioned just below the water surface next to the feed outlet and oriented vertically downward, providing a wide field of view covering most of the cage that allowed reliable monitoring of pellet consumption. The feeder system was previously tested to ensure its reliable and consistent operation. Then, for each delivered pellet, it was recorded which fish (if any) consumed it (images and a video recording of the feeder can be found in the repository https://github.com/JTomasFerrer/fish_ingestion_rates_JTFetal2025).

The single daily meal was monitored in 48 feeding trials (8 trials per cage, in the course of 4 months). Provided that the primary interest is to estimate the individual ingestion rate, the ration (number of pellets) delivered during each one of the 48 feeding trials was randomly set between 10 and 80% of the planned ration for the given cage and day, in order to reduce resource predictability, since it may cause dominant fish monopolising an even greater share of feed [63], thus conditioning the feeding behaviour of all the fish in a group. Nevertheless, the number of pellets delivered in any given feeding trial (ration, *R*) were recorded in order to test its effects on ingestion rate. Thus, note that two different variables related with feed availability were considered: the percentage of pellets delivered over the planned ration during a feeding trial (ration, *R*) and the number of pellets delivered according to the long-term diet level (diet, *D*).

Sea surface temperature was recorded daily (*Pendant Temperature/Light 64K Data Logger, HOBO*). Provided that the experimental sea cages are located very close to the navigation channel of a crowded marina, the number of motorised boats passing through the channel during the feeding trials time span on the days when feeding trials were performed was recorded as a proxy for anthropogenic stress [64].

### Statistical analysis

#### Modelling ingestion rate.

Functional response theory predicts that ingestion rate at time *t* depends on the amount of feed consumed till *t* [65]. At the time scale of meal duration, feeding dynamics models predict that, when an organism is fed *ad libitum*, ingestion rate will decrease as the stomach is filling [66]. After it, ingestion rate should be in equilibrium with the handling time (the average time spent on fully processing a feed item). However, a preliminary inspection of the data obtained (S1 Fig), together with preliminary modelling attempts in which formulations including ingestion rate slowdown failed to converge whereas linear formulations did, suggests that, despite some stochasticity, no apparent slowdown in ingestion rate was observed, suggesting that maximal stomach capacity (i.e., satiation) is not reached with our experimental settings. Therefore, ingestion rate could be treated as constant during the feeding trials, assuming satiation is not attained, as in [67].

Accordingly, the probability (*prob*) that the *i* fish from the replicate *j* of the cage *c* eats a given pellet is assumed to be the same at any moment of the feeding trial. The value of *prob*_*i,j,c*_ is given by the *softmax* mapping [68] of the score *sco*_*i,j,c*_, which is equivalent to the following equation:


$${prob}_{i,j,c}=\;\frac{e^{-{sco}_{i,j,c}}}{\sum_{i=\text{1}}^{i=\text{9}}e^{-{sco}_{i,j,c}}}$$ (1)

Where *i* = 1–8 denotes the eight fish in a cage and *i* = 9 corresponds to the case when the pellet is not consumed. The score *sco*_*i,j,c*_ is given by a lineal combination of body size, temperature, stress, diet, ration, fish, and replicate (Eq. 3), where more positive values denote a greater willingness of a given fish (and of a given cage and replicate) to consume a given feed pellet. Regarding fish size, [13] bioenergetic theory proposes that ingestion and assimilation rates are proportional to the organism’s surface. Therefore, we use the squared structural length – that is, the fish structural surface – (*L*^*2*^*,* units: cm^2^) as proxy of body size:


$$\overset{\text{2}}{\underset{i}{L}}={\left({LT}_{i}\;\delta \right)}^{\text{2}}$$ (2)

Where *LT* is the total length of the *i* fish (in cm) and *δ* is a species-specific shape coefficient, which for *D. labrax* has a value of 0.148 [69].

Temperature (*T*) effects on many physiological processes are expected to be unimodal, peaking at a given temperature and decreasing both at lower and higher temperatures [33]. Provided that the temperatures experienced by the fish are close to those many physiological traits seem to peak in *D. labrax* (e.g., growth rate peaks between 24 and 28 ºC [70–73]), for the sake of simplicity, here we assume a simple parabolic model because severe departures from mechanistic models (e.g., [73]) are only expected at low and high temperatures.

The number of boats passing close to the cages during the feeding trials was used as a proxy for anthropogenic stress (*S*). Diet level (*D;* 3 discrete categories: 60, 75 and 90%, where *D* takes a single categorical level according to the fish’s assigned diet level) and delivered ration size (*R*) effects were also included into the model, although not as parameters of primary interest but as covariables their putative effects should be accounted for.

Size-independent, fish-specific (*Fish*_*i,c*_) effects were modelled as a normally distributed random effect with zero mean and a between-fish standard deviation (*σ*_*Fish*_). Similarly, the variability between replicated feeding trials was modelled as a random effect with a between-replicate standard deviation (*σ*_*Replicate*_). Therefore:


$${sco}_{i,j.c}=\;\beta_{\text{0},\text{D}(\text{60},\text{75},\text{90}\%)}+\;\beta_{{\text{L}}^{\text{2}}}\overset{\text{2}}{\underset{i,c}{L}}+\beta_{\text{T}}T_{j,c}+\beta_{{\text{T}}^{\text{2}}}\overset{\text{2}}{\underset{j,c}{T}}+\beta_{\text{S}}S_{j,c}+\;\beta_{\text{R}}R_{j,c}+\;{Fish}_{i,c}+{Replicate}_{j,c}$$ (3)

For preventing overfitting, the score corresponding to the probability that a pellet was not consumed (*sco*_*i,j,c*_) is defined as:


$${sco}_{nc,j.c}=\;-\sum_{i=\text{1}}^{i=\text{8}}{sco}_{i,j,c}$$ (4)

Finally, the actual observation (i.e., which fish has consumed each one of the pellets delivered along each feeding trial) was considered a random realization of a multinomial distribution with a probability vector given by $prob=({prob}_{i=\text{1}\;to\;\text{8}},\;{prob}_{nc})$.

#### Estimating model parameters.

The model parameters (Eqs. 1–4) were estimated using a Bayesian approach. Advantages of the Bayesian approach are detailed elsewhere (e.g., [74]). Samples from the joint posterior distribution for the model parameters, given the data, were obtained using STAN [75] and *cmdstanr* library [76] of the R package [77]. Continuous explanatory variables were standardised (mean = 0.0, sd = 1.0) prior to the analysis. Weak informative priors were supplied for all parameters. The exact priors (both, distribution and parameters) are fully detailed in the code, which is available at https://github.com/JTomasFerrer/fish_ingestion_rates_JTFetal2025 to ensure transparency and reproducibility. Convergence was assessed visually and from the Gelman-Rubin diagnostic [78]. Posterior distribution of the parameters was estimated from 3 chains, 2,000 iterations each after 2,000 warm-up iterations [79].

#### Animal welfare statement.

All animal care procedures were approved by the Ethical Committee of Animal Experimentation of the University of the Balearic Islands (CEEA-UIB, ref. 153/12/20) and authorized by the Department of Environment, Agriculture and Fisheries of the Government of the Balearic Islands (ref. 2021/17/AEXP). All the procedures were carried out by trained competent personnel, in accordance with European Directive 2010/63/UE and Spanish Royal Decree RD53/2013 to ensure good practices for animal care, health and welfare.

## Results

### Between-individual variability in feed intake

A substantial and consistent between-individual variability in feed intake rate was observed across all cages and diet levels. The variance attributable to fish-specific effects *(σ*^*2*^_*F*_ = 0.431, Table 1) was markedly higher than the variance between repeated feeding trials (*σ*^*2*^_*R*_ = 0.183), with the fish-specific differences accounting for 70.2% of the residual variance *σ*^*2*^_*F*_ */ (σ*^*2*^_*F*_ + *σ*^*2*^_*R*_*).* This indicates that most of the unexplained variability in ingestion rate is driven by stable intrinsic fish-specificities differences (independent of the influences of body size and all other external variables), rather than differences among feeding trials. Moreover, such a large percentage strongly suggests that fish tended to consume a similar feed share across replicates throughout the duration of the experiment. Potential changes in hierarchy over time would be expected to manifest as increased variability between feeding trials and a reduced proportion of variance attributable to fish-specific effects.

**Table 1:** STAN selected result values for the different estimated variables in the stress-based model, including the median and the 95% CI posterior distributions.

Variable	Name	Q05	Median	Q95
β	General intercept	0.065	0.266	0.484
$\beta_{F}\;\sigma$	Between-fish standard deviation	0.362	0.431	0.526
$\beta_{R}\;\sigma$	Between-replicates standard deviation	0.138	0.183	0.249
$\beta_{L^{\text{2}}}$	Slope for size effect	−0.016	0.101	0.210
$\beta_{S}$	Slope for stress effect	−0.170	−0.104	−0.049
$\beta_{Diet\text{ 60}\%}$	Intercept for 60% diet level	0	0	0
$\beta_{Diet\text{ 75}\%}$	Intercept for 75% diet level	−0.369	−0.072	0.222
$\beta_{Diet\text{ 90}\%}$	Intercept for 90% diet level	−0.458	−0.136	0.151
$\beta_{R}$	Slope for ration size effect	−0.119	−0.050	0.013

**Fig 2:**
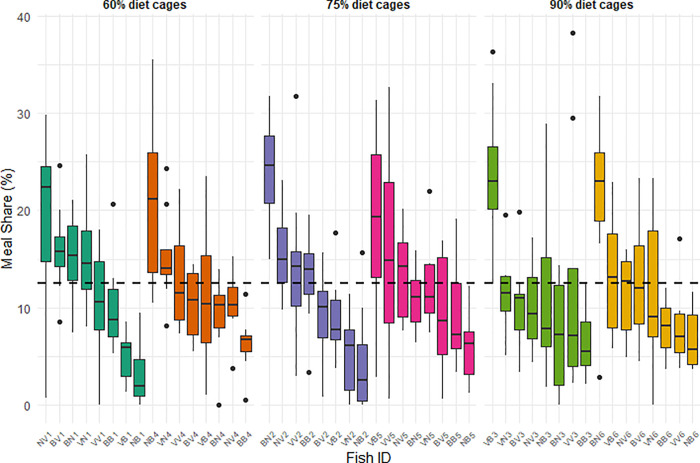
Individual meal shares distribution of the 48 experimental fish. The box plot for each fish shows the median (black line), interquantile range (box) and 95% range excluding outliers (whiskers); outliers are plotted as individual dots. Fish are grouped according to their diet level **(D)**, each colour representing a cage, and sorted by decreasing median meal share. The dashed line at 12.5% represents the expected share if feed was evenly distributed among the 8 fish of each cage.

This substantial between-fish variability in the observed individual meal share distribution is clearly visible (Fig 2). Some individuals consistently monopolised a large proportion of the feed resources, whereas others consistently consumed less than the expected 12.5% share (8 fish/cage), assuming no feed waste. These hierarchical patterns were similar across cages and diet treatments. Moreover, most fish maintained relatively consistent meal shares across trials compared to the magnitude of between-fish differences, reinforcing the temporal stability of these feeding patterns.

Additionally, feed waste was low in all treatments, with non-consumed pellets representing 4.9% in the 60% diet, 4.2% in the 75% diet, and 6.7% in the 90% diet, indicating that the observed variability was not driven by unequal feed availability but by differential access or feeding success among individuals.

**Fig 3:**
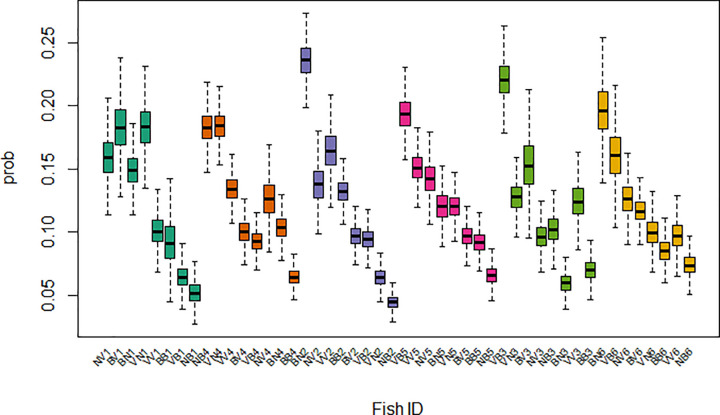
β_F_ (fish-specific variability after removing the effects of size and external variables). The box plot for each of the 48 fish show the median (black line) of β_F_ posterior distribution, the interquantile range (box) and the 95% range excluding outliers (whiskers). Fish are grouped by diet level (D) and cage, and sorted by decreasing median meal share, for consistency with Fig 2.

A similar pattern emerged from the model-derived fish-specific effects (*β*_*F*_). Fig 3 shows the probability of eating a pellet for each fish, assuming equal body size, stress level, and diet across all fish. These probabilities are directly derived from the fish-specific effects in Equation 3 (*β*_*F*_ values, S2 Table), which correspond to the fish-specificities independent of the influences of body size and all other external variables. These two patterns are consistent with the existence of well-defined hierarchies of feeding behaviour, which are stable in time and persist after statistically accounting for body size, stress, and feed availability.

### Model structure and selection

Preliminary inspection of the correlation pattern between the explanatory variables evidenced that temperature and anthropogenic stress (number of boats passing close to the cages) was highly correlated (r = 0.83) since temperature and touristic activity display close patterns in the Balearic Islands. Accordingly, the potential effects of these two variables on feeding cannot be disentangled. Therefore, two models were evaluated, each including all variables from equation 3 except for one, either temperature or stress. Both models converged satisfactorily, with all parameters exhibiting *rhat* values close to 1 and effective sample sizes well above standard thresholds (S1 and S3 Tables). Model comparison using leave-one-out cross-validation [80] indicates that the predictive accuracy of the two models falls well within the range of uncertainty (Table S4). Thus, the two models are indistinguishable from the statistical side.

However, the model prediction for temperature effects on ingestion rate suggest an unrealistic pattern, inconsistent with the biological expectations: feeding probability is predicted to decrease along the full range of temperatures actually experienced during the experiment (21.8 and 27.4 ºC), thus the model estimation of the optimal temperature for feeding should be under 21 ºC (S2 Fig). Instead, the optimal thermal range for *D. labrax* has been reported to be between 24 and 27 ºC for many metabolic and physiological processes [81–85]. Accordingly, the model including the anthropogenic stress proxy (number of boats passing close to the cages) was considered the most plausible and interpretable representation of the underlying processes. Therefore, all results and interpretations hereafter refer to the stress model only.

### Predictive performance of the selected model

**Fig 4:**
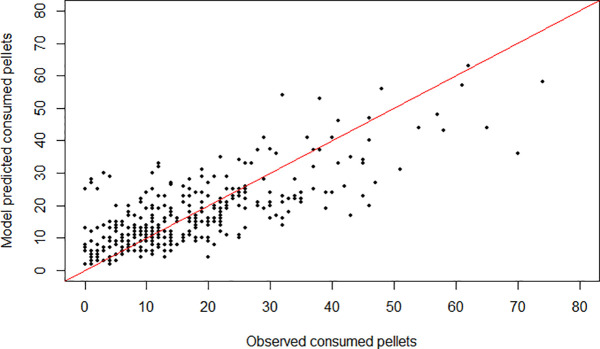
Comparison between observations vs model expectations for pellet consumption by individual fish. Each dot represents the actual number of pellets consumed by a fish during a meal (x-axis) and the corresponding model prediction (y-axis). The solid diagonal line represents the 1:1 relationship between predicted and observed values.

Regarding predictive accuracy, the actual number of pellets consumed by each fish at each trial is relatively well predicted by the model (Fig 4), considering the large stochasticity inherent to the feeding process [86].

The parameters estimations for the stress model are summarized in Table 1.

### Effects of intrinsic and extrinsic drivers

**Fig 5:**
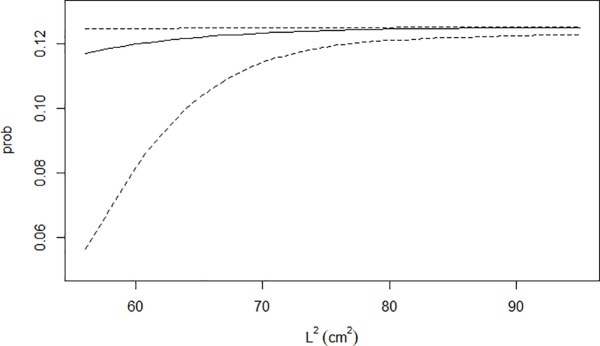
Effect of fish structural surface (L^2^) on the probability of pellet consumption. The x-axis represents the experimental range of L^2^. The y-axis shows the estimated probability of an individual fish consuming a given pellet, assuming all 8 fish in the cage are clones with the same body size, while all external variables are fixed. The solid line indicates the model-predicted median probability of consumption, while the dashed lines represent the 5th and 95th percentiles of the posterior distribution.

Regarding the effects of body size, stress and feed availability, they are all small or even not relevant. Therefore, it is not surprising that fish-specific *β*_*F*_ values are strongly correlated with the median of the actually observed meal shares (r = 0.89; S3 Fig). The median for the slope of body size effects in equation 3 (*β*_*L2*_) is positive (Table 1), indicating a positive effect of size on the probability that a fish eats a given pellet. However, the 5–95% quantile distribution for *β*_*L2*_ includes zero, suggesting that size effect on fish ingestion rate is not relevant. The expected effects of body size being all other constant are depicted in Fig 5, which reinforces the irrelevance of body size effects within the actual range of the fish included in the experiments. Obviously, it cannot be excluded that feeding behaviour can be affected by body size when smaller fish would be included in the experimental groups.

**Fig 6:**
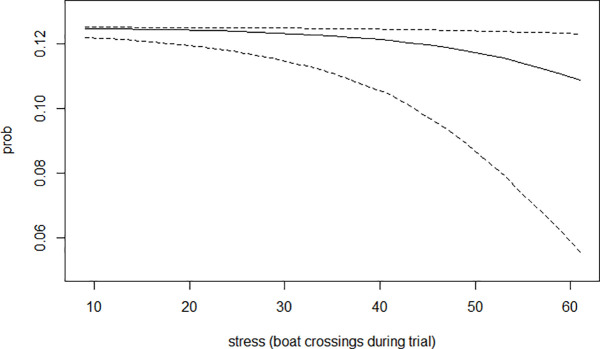
Effect of boat noise-induced stress on the probability of pellet consumption. The x-axis represents the number of boats passing close to the cage during the feeding trial, as a proxy for stress. The y-axis shows the estimated probability of an individual fish consuming a given pellet, assuming all 8 fish in the cage are clones with the same body size, while all other external variables are fixed. The solid line indicates the model-predicted median probability of consumption, while the dashed lines represent the 5th and 95th percentiles of the posterior distribution.

In contrast, the slope corresponding to stress (*β*_*S*_) (Table 1) shows a clearer negative impact on *prob*, indicating that environmental anthropogenic disturbances during the feeding trials reduced the feed intake rate (Fig 6).

Finally, neither the diet level (*β*_*D60,*_ *β*_*D75,*_ *β*_*D90*_) nor the feeding ration supplied in each feeding trial (*β*_*R*_) showed a relevant effect on the ingestion rate (Table 1). The three intercepts for diet level largely overlap, suggesting that the differences in the amount of feed delivered had no relevant effects on fish feeding, at least within the experimental range (60–90% of the recommended daily ration). Regarding the effect of the actually delivered ration at any given feeding trial, *β*_*R*_ was slightly negative but the 95% credibility interval includes zero. This result suggests that while fish are consuming more feed throughout a feeding trial, their feeding intake rate slightly decreases, albeit this decrease is not relevant within the observed range. This result reinforces the *a priori* assumption that feed intake rate remained the same during a given experimental trial, with no clear evidence of a slowdown that could be attributed to satiation (S4 Fig).

## Discussion

The fact that fish-specific effects accounted for more than 70% of the residual variance in ingestion rate, underscores the importance of persistent individual specificities in shaping feeding dynamics. This high proportion indicates that variability among fish largely exceeded short-term stochastic fluctuations between trials, suggesting strong temporal consistency in individual feeding behaviour. Rather than being fully explained by body size, stress, or feed availability, a substantial component of ingestion variability remained attributable to intrinsic fish-specific traits.

The strong correlation between the fish-specific effects (*β*_*F*_) and the empirically observed median meal shares further reinforces this interpretation. Since *β*_*F*_ values represent individual variability after statistically accounting for body size, stress, and feed availability, their close alignment with observed feeding patterns suggests that the modelling framework successfully isolated persistent intrinsic differences among fish.

Substantial between-individual variability in fish feed intake [8,14,16,87] and behaviour [88] has been widely reported, even among genetically similar individuals reared in identical conditions. Consistent with this, both the observed meal shares and the consumption probabilities inferred by our Bayesian model revealed substantial between-individual differences in ingestion rates. Notably, these differences persisted beyond the explanatory power of body size, anthropogenic stress, or feed availability, suggesting the presence of stable, intrinsic traits shaping feeding dynamics, emphasizing the need for assessment and management approaches that move beyond population averages and explicitly account for individual-level differences.

One likely mechanism behind this variation is the expression of social hierarchies, even in the absence of explicit aggressive behaviours. Subtle dominance-submission relationships may determine access to feed, feeding latency, or competition avoidance strategies, leading to consistent individual differences [15,16,54,89]. In this line, individual metabolic needs and behavioural traits could underlie the persistent differences in feeding behaviour [90–92]. In practical terms for aquaculture, this between-individual variability can lead to suboptimal feed efficiency, where dominant fish overeat and subordinates underperform, which ultimately will promote cohort size dispersion.

This study investigated the between-individual variability in feed intake rates in *D. labrax* under controlled experimental conditions, with the specific goal of disentangling intrinsic, fish-specific factors from the effects of variables previously identified as potential drivers of feeding behaviour, namely, body size, temperature, stress and feed availability. Between-fish differences in body size and growth have been usually attributed to differences in feed intake [10,43] which is considered a primary driver of growth [33]. Contrary to these expectations, our results suggest that the role of body size in determining feeding behaviour and shaping feeding hierarchies, although showing a positive effect, this is subtle within the observed range, and does not account for the substantial between-individual variability observed. Therefore, body size alone does not appear sufficient to explain the observed feeding hierarchies. In our case, fish with higher ingestion rates were not necessarily the largest, and other unaccounted intrinsic factors seems to play a crucial role. This result aligns with the observations of [52], who emphasizes the importance of energetic efficiency rather than body size itself; and those of [45] who found that body size, although is generally a good predictor of feed intake, did not influence individual feeding success in perch, pointing to other intrinsic factors (fish morphology, behaviour and metabolism) as potential drivers. The role of intrinsic factors other than size has also been suggested by [93]. Therefore, the relationship between ingestion rate and body size should still be considered an elusive topic.

In any case, the lack of relationship between ingestion rate and body size reported here leaves an open research question: Where does the surplus energy go in fish with higher feed ingestion if it is not allocated to growth? Here we suggest two non-mutually exclusive hypotheses. First, individual-specific differences in the energetic costs related with maintaining the social status within a hierarchy may drive that the effective energy input could be smaller than the expected one from the actually ingested feed amount [50,52]. Nevertheless, evidence is contradictory. For example, it has also been hypothesised that individual at lower social status would reduce feed consumption, metabolic activity and growth in order to prevent conflict [94]. Moreover, it has reported that individual at lower social status tend to display higher level of stress bioindicators, which in turn has been related with appetite inhibition [36,37].

Second, individual-specific differences in the way reserve energy is mobilized may drive to opposite life history strategies, even with similar energy input [11]. For example, some fish may display smaller mobilization rate of the reserve energy, which outputs in less energy available for growth and results in heavier and probably more resilient, but smaller individuals. The existence of such individual specificities in the strategies of energy management would be better explored by bioenergetic modelling, where frameworks like the one provided by the Dynamic Energy Budget theory [13] are particularly well-suited for this task, as they provide a formal structure to quantify how energy is assimilated, stored, mobilized, and allocated to maintenance, growth, and reproduction. In this context, measurements of the feed intake at the fish level, like those reported here are essential for accurate assessment of energy budgets [95].

Regarding the effects of temperature, the expectation is that ingestion rate should increase till reaching an optimal temperature, which, for many metabolic and physiological processes of the *D. labrax*, should be around 24–28 ºC [84,85,96]. However, in our case, the temperature actually experienced by the fish is highly correlated with anthropogenic stress. Thus, the specific effects of temperature cannot be disentangled from those of stress. Certainly, the model including temperature passed all the standard statistical quality controls and suggests a relevant effect of temperature. However, this model was discarded due to its lack of biological consistency. Our reasoning when selecting the model excluding temperature and including stress was not only to maximise the model predictive power, which is similar in both cases, but to select the model offering a sensible explanation of the biological processes behind. Thus, in our case, model selection was guided by the dilemma between quantitative *vs* qualitative fit [97]. Given the widely reported temperature-dependence pattern of other physiological and metabolic processes in seabass, a negative impact of temperature on ingestion rate from less than 21ºC onwards is considered unlikely. Thus, we hypothesise that the apparent effect of temperature reported here is an artefact resulting from collinearity of temperature with anthropogenic stress. However, note that we are not proposing that ingestion rate is, in general, temperature independent but that, in our case, any temperature effect is blurred by the stronger effects of stress on ingestion rate.

In any case, our results reinforce the role of stress as a driver capable to negatively impact on fish feeding, indicating an association of stress with reduced appetite, as proposed by other authors [36,89,98]. These findings raise awareness of the central role of animal welfare in aquaculture facilities [99], since it can affect production outcomes by reducing feed ingestion, and indeed a potential feed waste and its ecological consequences [100]. Further research should aim to comprehend deeper the effects of stress in fish feeding and whether differences in stress coping styles modulate the impact on feeding dynamics. In addition, the model does not account for potential acclimation to stress over time, as disentangling such effects would require independent control of correlated variables.

While diet level (60–90% of the recommended ration) did not significantly affect ingestion rate, this should not be interpreted as implying that total energy intake is equivalent across rations. Rather, within the experimental range, fish consumed pellets at a similar rate once feeding was initiated, regardless of the total ration supplied. Importantly, our analyses focused on ingestion dynamics during the initial phase of feeding. Therefore, the absence of diet effects on fish-specific ingestion rates reflects similar short-term ingestion rates across rations but does not allow inference about competitive processes over the full duration of the meal. Competitive asymmetries, if present, are more likely to influence cumulative intake as feeding progresses, particularly under restricted rations. Dedicated experiments explicitly targeting the complete feeding sequence would be required to evaluate such longer-term competitive effects.

Ingestion rate remained relatively stable along the four months duration of the experiment, and it was not affected by the ration actually delivered in each feeding trial, which varied within a large range (10–80% of the planned ration for each cage and day, which in addition may be between 60 and 90% of the feed manufacturer’s guidelines). This finding supports that fish do not seem to reach satiation because no sign of slowdown of ingestion rate was detected. This result is consistent with the low ratios of feed wasted reported here (4.2 to 6.7%), which is especially important in cage-based marine farming where fish are fed using sinking extruded or pelleted feed, as the fish have a limited time window to capture and consume the pellets before it passes through the bottom net at the cage base and is wasted [101].

Provided that satiation is not reached and ingestion rate can be considered constant within the rations actually delivered, the total amount of feed consumed by fish can be readily estimated from the fish-specific probability of pellet consumption and the total cage ration. This may be of applied interest in aquaculture, as taking individual fish as the unit of analysis enables the design of detailed guidelines for refining feeding and production techniques [102]. Moreover, in depth examination of feeding behaviour and its physiological consequences requires individual consumption rates to be known [18]. However, not reaching satiation also evidences a limitation of the experimental setting in our case: the maximum amount of feed each fish can consume when fed *at libitum* remains unknown. Estimating maximum consumption would require longer feeding trials and models allowing variable ingestion rate when approaching satiation [66].

Since the present study was conducted exclusively on female fish, potential sex-specific differences in feed ingestion rates were not assessed, which could represent an additional source of variability in feeding hierarchies that deserves future investigation.

The model included only main effects and did not test interaction terms; future studies should examine whether individual fish differ in their response to ration, potentially revealing more nuanced feeding dynamics.

Moreover, the feeding protocol used in the experiments differs from standard aquaculture feeding practices, representing another limitation of the experimental design. Since feed pellets were delivered sequentially at a slow rate, competition for feed access between fish may have enhanced feeding hierarchies compared to standard aquaculture feeding conditions. The relatively constant ingestion rate observed during these trials should therefore not be interpreted as evidence that fish were feeding at their physiological maximum rate but as fast as the automatic feeder delivered the feed, as far as satiation is not reached. Under near-satiation conditions, ingestion rate would be expected to decrease progressively.

Although pellet consumption is inherently stochastic at short time scales, the persistence of strong fish-specific effects despite this variability suggests that the observed feeding hierarchies reflect stable intrinsic differences rather than random fluctuations.

## Conclusions

In summary, we reported a considerable range of between-individual variability in feed ingestion rates in a group of fish reared in the same conditions. These between-fish differences, largely caused by fish-specific characteristics reinforces the relevance of fish personalities [103,104]. Moreover, these differences may be driven by different strategies of energy acquisition and allocation, which ultimately could result in divergent life histories that, in turn, implies a wide spectrum of outcomes with potential consequences for ecology and aquaculture. Finally, the results reported here underscore the limitations of assuming uniformity within fish cohorts and highlight the value of individualized approaches to feeding management [14]. These findings may also inform selective breeding strategies aimed at enhancing feed efficiency, resilience, and growth consistency in *D. labrax* populations. A better monitoring and understanding of the individual variability in feeding behaviour could help to refine feeding practices in the context of precision aquaculture [105]. In addition, the observed effects of stress on fish feeding behaviour highlights the need for proactive management strategies that minimize stressors and support the development of welfare-oriented indicators in aquaculture monitoring. Looking ahead, while video-monitoring of externally tagged fish cannot be readily scaled to commercial farming, recent advances in computer vision and artificial intelligence [106,107] show promise for real-time monitoring of between-individual variability in feed intake. Integrating these technological developments with mechanistic bioenergetic models, such as the DEB framework, may offer a powerful path forward to predict, manage, and ultimately optimize individual performance in aquaculture systems, as well as to a better understanding of ecological processes in wild populations.

## Supporting information

S1 Fig Observed feed ingestion rate of the 8 fish during a given feeding trial (cage 6, replicate 4). Solid lines represent the cumulated consumed pellets across time, while the dashed lines represent a constant feeding rate.
(TIF)

S2 Fig Effect of temperature on the probability of pellet consumption (temperature-based model). The x-axis represents the all-year-round temperature range of the Port d’Andratx. The y-axis shows the estimated probability of an individual fish consuming a given pellet, assuming all 8 fish in the cage are clones with the same body size, while all other external variables are fixed. The solid line indicates the model-predicted median probability of consumption, while the dashed lines represent the 5th and 95th percentiles of the posterior distribution. The red dashed vertical lines indicate the actual temperature range during the experiment. The temperature value at which probability of pellet consumption maximises is 21.0 ºC. This pattern does not fit a logic biological explanation and probably is due to the temperature effect being correlated with anthropogenic stress.
(TIF)

S3 Fig Relationship between the individual-level median value βF and the corresponding meal share. Each point represents a single fish.
(TIF)

S4 Fig Effect of delivered ration size (in % of feed delivered) on the probability of pellet consumption. The x-axis represents the experimental range of delivered ration size. The y-axis shows the estimated probability of an individual fish consuming a given pellet, assuming all 8 fish in the cage are clones with the same body size, while all external variables are fixed. The solid line indicates the model-predicted median probability of consumption, while the dashed lines represent the 5th and 95th percentiles of the posterior distribution.
(TIF)

S1 Table STAN result values for the different estimated variables in the stress-based model, including the median and the 95% CI posterior distributions, rhat values and effective sample sizes. (DOCX)

S2 Table Median and 5% and 95% percentiles for the β_F_ estimated values of the 48 fish analysed, with and their structural size (L^2^) and median consumed meal share (MS). (DOCX)

S3 Table STAN result values for the different estimated variables in the temperature-based model, including the median and the 95% CI posterior distributions, rhat values and effective sample sizes. (DOCX)

S4 Table Summary statistics from approximate leave-one-out cross-validation (LOO) assessing the out-of-sample predictive accuracy of the fitted Bayesian models (stress-based: SB and temperature-based: TB). The expected log predictive density (elpd_loo) provides a measure of model fit, with higher values indicating better predictive performance. The effective number of parameters (p_loo) reflects model complexity, and the LOO Information Criterion (looic) facilitates comparison across models (lower is better). Standard errors quantify the uncertainty in these estimates.
(DOCX)

## References

[1] Bi R, Liu H. Effects of variability among individuals on zooplankton population dynamics under environmental conditions. Mar Ecol Prog Ser. 2017;564:9–28. doi: 10.3354/meps11967

[2] Richmond CE, Rose KA, Breitburg DL. Individual variability and environmental conditions: Effects on zooplankton cohort dynamics. Mar Ecol Prog Ser. 2013;486:59–78. doi: 10.3354/meps10418

[3] Fritschie KJ, Olden JD. Disentangling the influences of mean body size and size structure on ecosystem functioning: an example of nutrient recycling by a non-native crayfish. Ecol Evol. 2015;6(1):159–69. doi: 10.1002/ece3.1852 26811781 PMC4716502

[4] Sibly RM, Grimm V, Martin BT, Johnston ASA, Kułakowska K, Topping CJ, et al. Representing the acquisition and use of energy by individuals in agent‐based models of animal populations. Methods Ecol Evol. 2012;4(2):151–61. doi: 10.1111/2041-210x.12002

[5] Cam E, Link WA, Cooch EG, Monnat J-Y, Danchin E. Individual covariation in life-history traits: seeing the trees despite the forest. Am Nat. 2002;159(1):96–105. doi: 10.1086/324126 18707403

[6] Johne AS, Carter CG, Wotherspoon S, Hadley S, Symonds JE, Walker SP, et al. Modeling the effects of ration on individual growth of Oncorhynchus tshawytscha under controlled conditions. J Fish Biol. 2023;103(5):1003–14. doi: 10.1111/jfb.15499 37410553

[7] Leblanc CA, Horri K, Skúlason S, Benhaim D. Subtle temperature increase can interact with individual size and social context in shaping phenotypic traits of a coldwater fish. PLoS One. 2019;14(3):e0213061. doi: 10.1371/journal.pone.0213061 30917136 PMC6436715

[8] Mccarthy ID, Carter CG, Houlihan DF. The effect of feeding hierarchy on individual variability in daily feeding of rainbow trout, Oncorhynchus mykiss (Walbaum). Journal of Fish Biology. 1992;41(2):257–63. doi: 10.1111/j.1095-8649.1992.tb02655.x

[9] Carlson SM, Satterthwaite WH. Weakened portfolio effect in a collapsed salmon population complex. Can J Fish Aquat Sci. 2011;68(9):1579–89. doi: 10.1139/f2011-084

[10] Campeas A, Brun-Bellut J, Baras E, Kestemont P, Gardeur JN. Growth heterogeneity in rearing sea bass (Dicentrarchus labrax): test of hypothesis with an iterative energetic model. Animal. 2009;3(9):1299–307. doi: 10.1017/S1751731109004595 22444906

[11] Palmer M, Moro-Martínez I, Tomàs-Ferrer J, Grau A, López-Belluga MD, Herlin M. Assessing between-individual variability in bioenergetics modelling: opportunities, challenges, and potential applications. Ecol Modell. 2024;498(July). doi: 10.1016/j.ecolmodel.2024.110848

[12] Sarà G, Reid GK, Rinaldi A, Palmeri V, Troell M, Kooijman SALM. Growth and reproductive simulation of candidate shellfish species at fish cages in the Southern Mediterranean: Dynamic Energy Budget (DEB) modelling for integrated multi-trophic aquaculture. Aquaculture. 2012;324–325:259–66. doi: 10.1016/j.aquaculture.2011.10.042

[13] Kooijman SALM. Dynamic Energy Budget Theory for Metabolic Organisation. 3 ed. Cambridge University Press. 2010.

[14] Goodrich HR, Clark TD. Why do some fish grow faster than others?. Fish and Fisheries. 2023;24(5):796–811. doi: 10.1111/faf.12770

[15] Irwin S, O’Halloran J, FitzGerald RD. The relationship between individual consumption and growth in juvenile turbot, Scophthalmus maximus. Aquaculture. 2002;204(1–2):65–74. doi: 10.1016/s0044-8486(01)00641-x

[16] Carter CG, Purser GJ, Houlihan DF, Thomas P. The Effect of Decreased Ration on Feeding Hierarchies in Groups of Greenback Flounder (Rhombosolea Tapirina: Teleostei). J Mar Biol Ass. 1996;76(2):505–16. doi: 10.1017/s0025315400030708

[17] Difford GF, Hatlen B, Gjerde B, Heia K, Baeverfjord G, Norris A, et al. Validation and genetic parameters of the X-ray method for phenotyping individual feed intake in Atlantic salmon. Aquaculture. 2024;586:740738. doi: 10.1016/j.aquaculture.2024.740738

[18] McCarthy ID, Houlihan DF, Carter CG, Moutou K. Variation in individual food consumption rates of fish and its implications for the study of fish nutrition and physiology. Proc Nutr Soc. 1993;52(3):427–36. doi: 10.1079/pns19930083 8302884

[19] Wang N, Hayward RS, Noltie DB. Variation in food consumption, growth, and growth efficiency among juvenile hybrid sunfish held individually. Aquaculture. 1998;167(1–2):43–52. doi: 10.1016/s0044-8486(98)00299-3

[20] Batzina A, Drossos IP, Giannoudaki K, Karakatsouli N. Effects of size variability on individual growth and feeding behavior of European seabass. Appl Anim Behav Sci. 2020;225(February):104963. doi: 10.1016/j.applanim.2020.104963

[21] Huntingford FA, Metcalfe NB, Thorpe JE, Graham WD, Adams CE. Social dominance and body size in Atlantic salmon parr, Salmo solar L. J Fish Biol. 1990;36(6):877–81. doi: 10.1111/j.1095-8649.1990.tb05635.x

[22] Azevedo ML, Silva T, Soares F, Budaev S, Conceição LEC, Rønnestad I. Development and evaluation of reference feed intake models for meagre (Argyrosomus regius). Aquac Eng. 2025;110(January). doi: 10.1016/j.aquaeng.2025.102526

[23] Gill A. How feeding performance and energy intake change with a small increase in the body size of the three-spined stickleback. Journal of Fish Biology. 1996;48(5):878–90. doi: 10.1006/jfbi.1996.0088

[24] Imsland AK, Nilsen T, Folkvord A. Stochastic simulation of size variation in turbot: possible causes analysed with an individual‐based model. Journal of Fish Biology. 1998;53(2):237–58. doi: 10.1111/j.1095-8649.1998.tb00978.x

[25] Kooijman SALM. Social interactions can affect feeding behaviour of fish in tanks. Journal of Sea Research. 2009;62(2–3):175–8. doi: 10.1016/j.seares.2009.06.003

[26] Wade NM, Clark TD, Maynard BT, Atherton S, Wilkinson RJ, Smullen RP, et al. Effects of an unprecedented summer heatwave on the growth performance, flesh colour and plasma biochemistry of marine cage-farmed Atlantic salmon (Salmo salar). J Therm Biol. 2019;80:64–74. doi: 10.1016/j.jtherbio.2018.12.021 30784489

[27] Stavrakidis-Zachou O, Lika K, Michail P, Tsalafouta A, Mohamed AH, Nikos P. Thermal tolerance, metabolic scope and performance of meagre, Argyrosomus regius, reared under high water temperatures. J Therm Biol. 2021;100:103063. doi: 10.1016/j.jtherbio.2021.103063 34503801

[28] Volkoff H, Rønnestad I. Effects of temperature on feeding and digestive processes in fish. Temperature (Austin). 2020;7(4):307–20. doi: 10.1080/23328940.2020.1765950 33251280 PMC7678922

[29] Handeland SO, Imsland AK, Stefansson SO. The effect of temperature and fish size on growth, feed intake, food conversion efficiency and stomach evacuation rate of Atlantic salmon post-smolts. Aquaculture. 2008;283(1–4):36–42. doi: 10.1016/j.aquaculture.2008.06.042

[30] Føre M, Alver M, Alfredsen JA, Marafioti G, Senneset G, Birkevold J, et al. Modelling growth performance and feeding behaviour of Atlantic salmon (Salmo salar L.) in commercial-size aquaculture net pens: Model details and validation through full-scale experiments. Aquaculture. 2016;464:268–78. doi: 10.1016/j.aquaculture.2016.06.045 28148974 PMC5268353

[31] Oppedal F, Dempster T, Stien LH. Environmental drivers of Atlantic salmon behaviour in sea-cages: A review. Aquaculture. 2011;311(1–4):1–18. doi: 10.1016/j.aquaculture.2010.11.020

[32] Nati JJH, Lindström J, Halsey LG, Killen SS. Is there a trade-off between peak performance and performance breadth across temperatures for aerobic scope in teleost fishes? Biol Lett. 2016;12(9):20160191. doi: 10.1098/rsbl.2016.0191 27677812 PMC5046912

[33] Assan D, Huang Y, Mustapha UF, Addah MN, Li G, Chen H. Fish Feed Intake, Feeding Behavior, and the Physiological Response of Apelin to Fasting and Refeeding. Front Endocrinol (Lausanne). 2021;12:798903. doi: 10.3389/fendo.2021.798903 34975769 PMC8715717

[34] Madison BN, Tavakoli S, Kramer S, Bernier NJ. Chronic cortisol and the regulation of food intake and the endocrine growth axis in rainbow trout. J Endocrinol. 2015;226(2):103–19. doi: 10.1530/JOE-15-0186 26101374

[35] Dambrine C, Huret M, Woillez M, Pecquerie L, Allal F, Servili A. Contribution of a bioenergetics model to investigate the growth and survival of European seabass in the Bay of Biscay – English Channel area. Ecol Modell. 2020;423(August 2019):109007. doi: 10.1016/j.ecolmodel.2020.109007

[36] Bernier NJ. The corticotropin-releasing factor system as a mediator of the appetite-suppressing effects of stress in fish. Gen Comp Endocrinol. 2006;146(1):45–55. doi: 10.1016/j.ygcen.2005.11.016 16410007

[37] Leal E, Fernández-Durán B, Guillot R, Ríos D, Cerdá-Reverter JM. Stress-induced effects on feeding behavior and growth performance of the sea bass (Dicentrarchus labrax): a self-feeding approach. J Comp Physiol B. 2011;181(8):1035–44. doi: 10.1007/s00360-011-0585-z 21594625

[38] Ashley PJ. Fish welfare: Current issues in aquaculture. Appl Anim Behav Sci. 2007;104(3–4):199–235. doi: 10.1016/j.applanim.2006.09.001

[39] Barcelo-Serra M, Cabanellas S, Palmer M, Bolgan M, Alós J. A state-space model to derive motorboat noise effects on fish movement from acoustic tracking data. Sci Rep. 2021;11(1):4765. doi: 10.1038/s41598-021-84261-2 33637805 PMC7910575

[40] Kim B, Jin G, Byeon Y, Park SY, Song H, Lee C, et al. Monitoring of the physiological responses of marine fishes to construction and operation noise from offshore wind farms. Mar Pollut Bull. 2025;218:118139. doi: 10.1016/j.marpolbul.2025.118139 40381444

[41] Besson M, Feeney WE, Moniz I, François L, Brooker RM, Holzer G, et al. Anthropogenic stressors impact fish sensory development and survival via thyroid disruption. Nat Commun. 2020;11(1):3614. doi: 10.1038/s41467-020-17450-8 32681015 PMC7367887

[42] Gilmour KM, Dibattista JD, Thomas JB. Physiological causes and consequences of social status in salmonid fish. Integr Comp Biol. 2005;45(2):263–73. doi: 10.1093/icb/45.2.263 21676770

[43] Azaza MS, Assad A, Maghrbi W, El-Cafsi M. The effects of rearing density on growth, size heterogeneity and inter-individual variation of feed intake in monosex male Nile tilapia Oreochromis niloticus L. Animal. 2013;7(11):1865–74. doi: 10.1017/S1751731113001493 23915501

[44] Jobling M, Reinsnes T‐G. Physiological and social constraints on growth of Arctic charr, Salvelinus alpinus L.: an investigation of factors leading to stunting. J Fish Biol. 1986;28(3):379–84. doi: 10.1111/j.1095-8649.1986.tb05174.x

[45] Staffan F, Magnhagen C, Alanärä A. Variation in food intake within groups of juvenile perch. Journal of Fish Biology. 2002;60(3):771–4. doi: 10.1111/j.1095-8649.2002.tb01702.x

[46] Jobling M, Covès D, Damsgård B, Kristiansen HR, Koskela J, Petursdottir TE, et al. Techniques for Measuring Feed Intake. In: Houlihan D, Boujard T, Jobling M, editors. Food Intake in Fish. Oxford: Blackwell Science Ltd. 2001:49–87.

[47] Besson M, Allal F, Chatain B, Vergnet A, Clota F, Vandeputte M. Combining Individual Phenotypes of Feed Intake With Genomic Data to Improve Feed Efficiency in Sea Bass. Front Genet. 2019;10:219. doi: 10.3389/fgene.2019.00219 30984235 PMC6449465

[48] Martins CIM, Conceição LEC, Schrama JW. Feeding behavior and stress response explain individual differences in feed efficiency in juveniles of Nile tilapia Oreochromis niloticus. Aquaculture. 2011;312(1–4):192–7. doi: 10.1016/j.aquaculture.2010.12.035

[49] Rodde C, Vandeputte M, Trinh TQ, Douchet V, Canonne M, Benzie JAH, et al. The Effects of Feed Restriction and Isolated or Group Rearing on the Measurement of Individual Feed Intake and Estimation of Feed Conversion Ratio in Juvenile Nile Tilapia (Oreochromis niloticus) for Selective Breeding Purposes. Front Genet. 2021;11:596521. doi: 10.3389/fgene.2020.596521 33519898 PMC7844319

[50] Silverstein JT. Relationships among feed intake, feed efficiency, and growth in juvenile rainbow trout. N Am J Aquac. 2006;68(2):168–75. doi: 10.1577/a05-010.1

[51] Carter CG, Houlihan DF, McCarthy ID, Brafield AE. Variation in the food intake of grass carp,Ctenopharyngodon idella(Val.), fed singly or in groups. Aquat Living Resour. 1992;5(3):225–8. doi: 10.1051/alr:1992022

[52] Elvy JE, Symonds JE, Hilton Z, Walker SP, Tremblay LA, Casanovas P, et al. The relationship of feed intake, growth, nutrient retention, and oxygen consumption to feed conversion ratio of farmed saltwater Chinook salmon (Oncorhynchus tshawytscha). Aquaculture. 2022;554:738184. doi: 10.1016/j.aquaculture.2022.738184

[53] Elvy JE, Symonds JE, Hilton Z, Walker SP, Tremblay LA, Casanovas P, et al. Determining differences in the timing of fish feed intake using a novel dual ballotini X-radiography method. Aquaculture. 2024;588:740883. doi: 10.1016/j.aquaculture.2024.740883

[54] McCarthy ID, Gair DJ, Houlihan DF. Feeding rank and dominance in Tilapia rendalli under defensible and indefensible patterns of food distribution. Journal of Fish Biology. 1999;55(4):854–67. doi: 10.1111/j.1095-8649.1999.tb00722.x

[55] Ahmad A, Sonesson AK, Hatlen B, Bæverfjord G, Berg P, Norris A, et al. Genetic analysis of individual feed intake and efficiency in Atlantic salmon smolts using X-ray imaging. Aquaculture. 2025;608:742715. doi: 10.1016/j.aquaculture.2025.742715

[56] de Verdal H, Mekkawy W, Lind CE, Vandeputte M, Chatain B, Benzie JAH. Measuring individual feed efficiency and its correlations with performance traits in Nile tilapia, Oreochromis niloticus. Aquaculture. 2017;468:489–95. doi: 10.1016/j.aquaculture.2016.11.015

[57] Øverli Ø, Winberg S, Damsgård B, Jobling M. Food intake and spontaneous swimming activity in Arctic char (Salvelinus alpinus): Role of brain serotonergic activity and social interactions. Can J Zool. 1998;76(7):1366–70. doi: 10.1139/z98-050

[58] Smith IP, Metcalfe NB, Huntingford FA. The effects of food pellet dimensions on feeding responses by Atlantic salmon (Salmo salar L.) in a marine net pen. Aquaculture. 1995;130(2–3):167–75. doi: 10.1016/0044-8486(94)00207-5

[59] FAO. Global production. Fisheries and Aquaculture. https://www.fao.org/fishery/en/collection/global_production?lang=en. 2016.

[60] Vandeputte M, Gagnaire P-A, Allal F. The European sea bass: a key marine fish model in the wild and in aquaculture. Anim Genet. 2019;50(3):195–206. doi: 10.1111/age.12779 30883830 PMC6593706

[61] Chatain B, Chavanne H. La génétique du bar (Dicentrarchus labrax L.). Cahiers Agricultures. 2009;18(2):249–55. doi: 10.1684/agr.2009.0296

[62] Stefánsson MÖ, Imsland AK, Jenssen MD, Jonassen TM, Stefansson SO, FitzGerald R. The effect of different initial size distributions on the growth of Atlantic halibut. Journal of Fish Biology. 2000;56(4):826–36. doi: 10.1111/j.1095-8649.2000.tb00875.x

[63] Grand TC, Grant JWA. Spatial predictability of food influences its monopolization and defence by juvenile convict cichlids. Animal Behaviour. 1994;47(1):91–100. doi: 10.1006/anbe.1994.1010

[64] Harding HR, Gordon TAC, Wong K, McCormick MI, Simpson SD, Radford AN. Condition-dependent responses of fish to motorboats: Condition-dependent responses to noise. Biol Lett. 2020;16(11):1–6. doi: 10.1098/rsbl.2020.0401 33202186 PMC7728680

[65] Rosenbaum B, Rall BC. Fitting functional responses: Direct parameter estimation by simulating differential equations. Methods Ecol Evol. 2018;9(10):2076–90. doi: 10.1111/2041-210x.13039

[66] Thomas DM, Paynter J, Peterson CM, Heymsfield SB, Nduati A, Apolzan JW, et al. A new universal dynamic model to describe eating rate and cumulative intake curves. Am J Clin Nutr. 2017;105(2):323–31. doi: 10.3945/ajcn.115.127811 28077377 PMC5267295

[67] Elliott JM, Persson L. The Estimation of Daily Rates of Food Consumption for Fish. The Journal of Animal Ecology. 1978;47(3):977. doi: 10.2307/3682

[68] Franke M, Degen J. The softmax function: Properties, motivation, and interpretation. PsyArXiv. 2023. 1–23.

[69] Stavrakidis-Zachou O, Papandroulakis N, Lika K. A DEB model for European sea bass (Dicentrarchus labrax): Parameterisation and application in aquaculture. Journal of Sea Research. 2019;143:262–71. doi: 10.1016/j.seares.2018.05.008

[70] Person-Le Ruyet J, Mahé K, Le Bayon N, Le Delliou H. Effects of temperature on growth and metabolism in a Mediterranean population of European sea bass, Dicentrarchus labrax. Aquaculture. 2004;237(1–4):269–80. doi: 10.1016/j.aquaculture.2004.04.021

[71] Stavrakidis-Zachou O, Lika K, Pavlidis M, Asaad MH, Papandroulakis N. Metabolic scope, performance and tolerance of juvenile European sea bass Dicentrarchus labrax upon acclimation to high temperatures. PLoS One. 2022;17(8):e0272510. doi: 10.1371/journal.pone.0272510 35960751 PMC9374223

[72] Islam MJ, Slater MJ, Bögner M, Zeytin S, Kunzmann A. Extreme ambient temperature effects in European seabass, Dicentrarchus labrax: Growth performance and hemato-biochemical parameters. Aquaculture. 2020;522:735093. doi: 10.1016/j.aquaculture.2020.735093

[73] Dell AI, Pawar S, Savage VM. Systematic variation in the temperature dependence of physiological and ecological traits. Proc Natl Acad Sci U S A. 2011;108(26):10591–6. doi: 10.1073/pnas.1015178108 21606358 PMC3127911

[74] Kruschke J. Doing Bayesian data analysis: A tutorial with R, JAGS, and Stan. 2014.

[75] Plummer M. Simulation-Based Bayesian Analysis. 2023:401–25.

[76] Gabry J, Češnovar R, Johnson A. cmdstanr: R Interface to “CmdStan”. 2023.

[77] R Core Team. Vienna, Austria: R Foundation for Statistical Computing. 2022.

[78] Vehtari A, Gelman A, Simpson D, Carpenter B, Bürkner P-C. Rank-Normalization, Folding, and Localization: An Improved Rˆ for Assessing Convergence of MCMC (with Discussion). Bayesian Anal. 2021;16(2). doi: 10.1214/20-ba1221

[79] Stan Development Team. CmdStan user’s guide, version 2.30.1. 2022.

[80] Vehtari A, Gabry J, Magnusson M, Yao Y, Bürkner PC, Paananen T. loo: Efficient leave-one-out cross-validation and WAIC for Bayesian models. The R Foundation. 2024.

[81] Abdel-Tawwab M, Omar AA, Khalil RH, Selema TAMA, Elsamanooudy SI, El-Saftawy HAM, et al. Influences of thermal stress on the growth biometrics, stress indicators, oxidative stress biomarkers, and histopathological alterations in European seabass, Dicentrarchus labrax, juveniles. Fish Physiol Biochem. 2025;51(2):70. doi: 10.1007/s10695-025-01470-6 40111646 PMC11926022

[82] Claridge PN, Potter IC. Movements, abundance, age composition and growth of bass, Dicentrarchus labrax, in the Severn Estuary and inner Bristol Channel. J Mar Biol Ass. 1983;63(4):871–9. doi: 10.1017/s0025315400071289

[83] Islam MJ, Kunzmann A, Bögner M, Meyer A, Thiele R, James Slater M. Metabolic and molecular stress responses of European seabass, Dicentrarchus labrax at low and high temperature extremes. Ecol Indic. 2020;112(10). doi: 10.1016/j.ecolind.2020.106118

[84] Person-Le Ruyet J, Mahé K, Le Bayon N, Le Delliou H. Effects of temperature on growth and metabolism in a Mediterranean population of European sea bass, Dicentrarchus labrax. Aquaculture. 2004;237(1–4):269–80. doi: 10.1016/j.aquaculture.2004.04.021

[85] Vinagre C, Madeira D, Narciso L, Cabral HN, Diniz M. Effect of temperature on oxidative stress in fish: lipid peroxidation and catalase activity in the muscle of juvenile seabass, Dicentrarchus labrax. Ecol Indic. 2012;23:274–9. doi: 10.1016/j.ecolind.2012.04.009

[86] Augustine S, Litvak MK, Kooijman SALM. Stochastic feeding of fish larvae and their metabolic handling of starvation. Journal of Sea Research. 2011;66(4):411–8. doi: 10.1016/j.seares.2011.07.006

[87] Metcalfe NB. Intraspecific variation in competitive ability and food intake in salmonids: consequences for energy budgets and growth rates. Journal of Fish Biology. 1986;28(5):525–31. doi: 10.1111/j.1095-8649.1986.tb05190.x

[88] Bierbach D, Laskowski KL, Wolf M. Behavioural individuality in clonal fish arises despite near-identical rearing conditions. Nat Commun. 2017;8:15361. doi: 10.1038/ncomms15361 28513582 PMC5442312

[89] Best C, Jennings K, Culbert BM, Flear K, Volkoff H, Gilmour KM. Too stressed to eat: Investigating factors associated with appetite loss in subordinate rainbow trout. Mol Cell Endocrinol. 2023;559:111798. doi: 10.1016/j.mce.2022.111798 36243201

[90] Dijkstra PD. Growth rate is associated with reduced oxidative stress and this effect is modulated by the degree of social dominance in males of an African cichlid fish. Comp Biochem Physiol A Mol Integr Physiol. 2025;307:111892. doi: 10.1016/j.cbpa.2025.111892 40490211 PMC12276911

[91] Réale D, Reader SM, Sol D, McDougall PT, Dingemanse NJ. Integrating animal temperament within ecology and evolution. Biol Rev Camb Philos Soc. 2007;82(2):291–318. doi: 10.1111/j.1469-185X.2007.00010.x 17437562

[92] Campos-Candela A, Palmer M, Balle S, Álvarez A, Alós J. A mechanistic theory of personality-dependent movement behaviour based on dynamic energy budgets. Ecol Lett. 2019;22(2):213–32. doi: 10.1111/ele.13187 30467933

[93] Henriksen R, Höglund A, Fogelholm J, Abbey-Lee R, Johnsson M, Dingemanse NJ, et al. Intra-Individual Behavioural Variability: A Trait under Genetic Control. Int J Mol Sci. 2020;21(21):8069. doi: 10.3390/ijms21218069 33138119 PMC7663371

[94] Wong MYL, Munday PL, Buston PM, Jones GP. Fasting or feasting in a fish social hierarchy. Curr Biol. 2008;18(9):R372–3. doi: 10.1016/j.cub.2008.02.063 18460314

[95] Doulah A, McCrory MA, Higgins JA, Sazonov E. A Systematic Review of Technology-Driven Methodologies for Estimation of Energy Intake. IEEE Access. 2019;7:49653–68. doi: 10.1109/access.2019.2910308 32489752 PMC7266287

[96] Stavrakidis-Zachou O, Lika K, Pavlidis M, Asaad MH, Papandroulakis N. Metabolic scope, performance and tolerance of juvenile European sea bass Dicentrarchus labrax upon acclimation to high temperatures. PLoS One. 2022;17(8):e0272510. doi: 10.1371/journal.pone.0272510 35960751 PMC9374223

[97] Navarro DJ. Between the Devil and the Deep Blue Sea: Tensions Between Scientific Judgement and Statistical Model Selection. Comput Brain Behav. 2018;2(1):28–34. doi: 10.1007/s42113-018-0019-z

[98] Conde-Sieira M, Chivite M, Míguez JM, Soengas JL. Stress Effects on the Mechanisms Regulating Appetite in Teleost Fish. Front Endocrinol (Lausanne). 2018;9:631. doi: 10.3389/fendo.2018.00631 30405535 PMC6205965

[99] Arechavala‐Lopez P, Cabrera‐Álvarez MJ, Maia CM, Saraiva JL. Environmental enrichment in fish aquaculture: A review of fundamental and practical aspects. Reviews in Aquaculture. 2021;14(2):704–28. doi: 10.1111/raq.12620

[100] Øverli Ø, Sørensen C, Kiessling A, Pottinger TG, Gjøen HM. Selection for improved stress tolerance in rainbow trout (Oncorhynchus mykiss) leads to reduced feed waste. Aquaculture. 2006;261(2):776–81. doi: 10.1016/j.aquaculture.2006.08.049

[101] Kousoulaki K, Sether BS, Albrektsen S, Noble C. Review on European sea bass (Dicentrarchus labrax, Linnaeus, 1758) nutrition and feed management: A practical guide for optimizing feed formulation and farming protocols. Aquac Nutr. 2015;21(2):129–51. doi: 10.1111/anu.12233

[102] Du Z, Cui M, Wang Q, Liu X, Xu X, Bai Z, et al. Feeding intensity assessment of aquaculture fish using Mel spectrogram and deep learning algorithms. Aquac Eng. 2023;102(February):102345. doi: 10.1016/j.aquaeng.2023.102345

[103] Réale D, Reader SM, Sol D, McDougall PT, Dingemanse NJ. Integrating animal temperament within ecology and evolution. Biol Rev Camb Philos Soc. 2007;82(2):291–318. doi: 10.1111/j.1469-185X.2007.00010.x 17437562

[104] Conrad JL, Weinersmith KL, Brodin T, Saltz JB, Sih A. Behavioural syndromes in fishes: a review with implications for ecology and fisheries management. J Fish Biol. 2011;78(2):395–435. doi: 10.1111/j.1095-8649.2010.02874.x 21284626

[105] Føre M, Frank K, Norton T, Svendsen E, Alfredsen JA, Dempster T. Precision fish farming: A new framework to improve production in aquaculture. Biosyst Eng. 2018;173:176–93. doi: 10.1016/j.biosystemseng.2017.10.014

[106] Cui M, Liu X, Liu H, Zhao J, Li D, Wang W. Fish tracking, counting, and behaviour analysis in digital aquaculture: A comprehensive review. 2025. 10.1111/raq.13001

[107] Feng M, Jiang P, Wang Y, Hu S, Chen S, Li R, et al. YOLO-feed: An advanced lightweight network enabling real-time, high-precision detection of feed pellets on CPU devices and its applications in quantifying individual fish feed intake. Aquaculture. 2025;608:742700. doi: 10.1016/j.aquaculture.2025.742700

