# Supplementary figures and images for: Size-independent, between-individual variability in feed ingestion rate in European seabass (Dicentrarchus labrax)
Source: PLoS One. 2026 Apr 16;21(4):e0347113. doi: 10.1371/journal.pone.0347113 (PMC13086339; doi:10.1371/journal.pone.0347113)

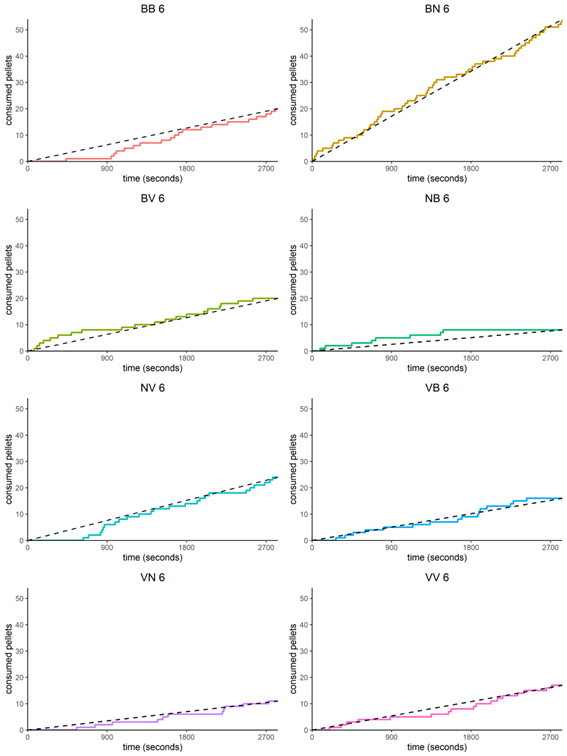

Supplement: S1 Fig — Solid lines represent the cumulated consumed pellets across time, while the dashed lines represent a constant feeding rate. (TIF) [file pone.0347113.s001.tif]
