# Supplementary material for: Size-independent, between-individual variability in feed ingestion rate in European seabass (Dicentrarchus labrax)
Source: PLoS One. 2026 Apr 16;21(4):e0347113. doi: 10.1371/journal.pone.0347113 (PMC13086339; doi:10.1371/journal.pone.0347113)
Supplement: S1 Table — (DOCX) [file pone.0347113.s005.docx]

Table S1: STAN result values for the different estimated variables in the stress-based model, including the median and the 95% CI posterior distributions, rhat values and effective sample sizes.

| Variable | Name | Q05 | Median | Q95 | *rhat* | ESS |
| --- | --- | --- | --- | --- | --- | --- |
| $\beta$ | General intercept | 0.065 | 0.266 | 0.484 | 1.0003 | 1320 |
| $\beta_{F} \sigma$ | Between-fish standard deviation | 0.362 | 0.431 | 0.526 | 1.0009 | 3092 |
| $\beta_{R} \sigma$ | Between-replicates standard deviation | 0.138 | 0.183 | 0.249 | 1.0013 | 1247 |
| $\beta_{L^{2}}$ | Slope for size effect | -0.016 | 0.101 | 0.210 | 1.0012 | 1500 |
| $\beta_{S}$ | Slope for stress effect | -0.170 | -0.104 | -0.049 | 1.0007 | 2439 |
| $\beta_{Diet 60\%}$ | Intercept for 60% diet level | 0 | 0 | 0 | - | - |
| $\beta_{Diet 75\%}$ | Intercept for 75% diet level | -0.369 | -0.072 | 0.222 | 1.0007 | 1158 |
| $\beta_{Diet 90\%}$ | Intercept for 90% diet level | -0.458 | -0.136 | 0.151 | 1.0015 | 1460 |
| $\beta_{R}$ | Slope for ration size effect | -0.119 | -0.050 | 0.013 | 1.0014 | 1853 |
