# Supplementary material for: Size-independent, between-individual variability in feed ingestion rate in European seabass (Dicentrarchus labrax)
Source: PLoS One. 2026 Apr 16;21(4):e0347113. doi: 10.1371/journal.pone.0347113 (PMC13086339; doi:10.1371/journal.pone.0347113)
Supplement: S2 Table — (DOCX) [file pone.0347113.s006.docx]

Table S2: Median and 5% and 95% percentiles for the β_F_ estimated values of the 48 fish analysed, with and their structural size (L^2^) and median consumed meal share (MS).

| **Fish** | **L2** | **β_F_ Q05** | **β_F_ median** | **β_F_ Q95** | **MS (%)** |
| --- | --- | --- | --- | --- | --- |
| BB1 | 95.363 | -0.59489 | -0.21443 | 0.15082 | 8.77 |
| BN1 | 69.567 | 0.01662 | 0.27918 | 0.54228 | 15.33 |
| BV1 | 63.043 | 0.17612 | 0.48257 | 0.76651 | 15.79 |
| NB1 | 63.515 | -1.15768 | -0.78539 | -0.42770 | 2.00 |
| NV1 | 81.153 | 0.07729 | 0.34349 | 0.60353 | 22.38 |
| VB1 | 68.089 | -0.88265 | -0.56600 | -0.25209 | 5.97 |
| VN1 | 64.942 | 0.20824 | 0.48806 | 0.75932 | 14.55 |
| VV1 | 79.292 | -0.38543 | -0.11371 | 0.15038 | 10.57 |
| BB2 | 53.377 | -0.07382 | 0.17091 | 0.41186 | 13.93 |
| BN2 | 77.714 | 0.51125 | 0.75102 | 0.98670 | 24.61 |
| BV2 | 70.81 | -0.39376 | -0.14000 | 0.10801 | 10.05 |
| NB2 | 79.821 | -1.22116 | -0.92384 | -0.63917 | 2.62 |
| NV2 | 85.213 | -0.07850 | 0.20952 | 0.50373 | 15.02 |
| VB2 | 70.311 | -0.41845 | -0.17116 | 0.08351 | 7.73 |
| VN2 | 70.062 | -0.83602 | -0.55884 | -0.29579 | 6.11 |
| VV2 | 64.942 | 0.13001 | 0.38420 | 0.64944 | 14.23 |
| BB3 | 76.671 | -0.74511 | -0.46868 | -0.18937 | 5.49 |
| BN3 | 72.822 | -0.92221 | -0.62253 | -0.34908 | 7.17 |
| BV3 | 58.872 | 0.02976 | 0.32145 | 0.60311 | 10.95 |
| NB3 | 68.58 | -0.34282 | -0.08396 | 0.17105 | 7.83 |
| NV3 | 78.238 | -0.40788 | -0.14382 | 0.13156 | 9.38 |
| VB3 | 74.862 | 0.44959 | 0.68770 | 0.92350 | 23.03 |
| VN3 | 75.377 | -0.11313 | 0.14197 | 0.38617 | 11.60 |
| VV3 | 81.957 | -0.17032 | 0.11289 | 0.39026 | 7.14 |
| BB4 | 78.501 | -0.83455 | -0.57212 | -0.31833 | 6.69 |
| BN4 | 70.311 | -0.34773 | -0.09782 | 0.14898 | 10.23 |
| BV4 | 83.06 | -0.38806 | -0.13031 | 0.13190 | 10.80 |
| NB4 | 82.226 | 0.23404 | 0.47462 | 0.71487 | 21.18 |
| NV4 | 63.989 | -0.18866 | 0.10228 | 0.37620 | 10.27 |
| VB4 | 81.957 | -0.47331 | -0.21546 | 0.04439 | 10.36 |
| VN4 | 74.094 | 0.25057 | 0.48182 | 0.70705 | 14.07 |
| VV4 | 79.821 | -0.07567 | 0.16163 | 0.40180 | 11.50 |
| BB5 | 73.329 | -0.47718 | -0.20624 | 0.04573 | 7.25 |
| BN5 | 65.181 | -0.20890 | 0.06134 | 0.33279 | 11.11 |
| BV5 | 73.075 | -0.41611 | -0.15610 | 0.09256 | 8.65 |
| NB5 | 67.113 | -0.82697 | -0.54693 | -0.27851 | 6.29 |
| NV5 | 63.752 | -0.03531 | 0.23006 | 0.49803 | 14.19 |
| VB5 | 75.119 | 0.30083 | 0.54180 | 0.77466 | 19.33 |
| VN5 | 72.822 | -0.18687 | 0.06297 | 0.30495 | 11.06 |
| VV5 | 74.094 | 0.04083 | 0.28930 | 0.52540 | 14.90 |
| BB6 | 62.338 | -0.55153 | -0.28976 | -0.02697 | 8.14 |
| BN6 | 80.353 | 0.29967 | 0.54694 | 0.81807 | 22.98 |
| BV6 | 72.064 | -0.20349 | 0.02675 | 0.27163 | 12.02 |
| NB6 | 62.3388 | -0.70403 | -0.43411 | -0.16452 | 5.71 |
| NV6 | 60.939 | -0.15307 | 0.10594 | 0.37644 | 12.70 |
| VB6 | 55.948 | 0.04950 | 0.34512 | 0.64805 | 13.11 |
| VN6 | 80.353 | -0.39483 | -0.12919 | 0.14814 | 9.14 |
| VV6 | 58.417 | -0.44340 | -0.15978 | 0.12372 | 6.98 |
