# Supplementary material for: Size-independent, between-individual variability in feed ingestion rate in European seabass (Dicentrarchus labrax)
Source: PLoS One. 2026 Apr 16;21(4):e0347113. doi: 10.1371/journal.pone.0347113 (PMC13086339; doi:10.1371/journal.pone.0347113)
Supplement: S3 Table — (DOCX) [file pone.0347113.s007.docx]

Table S3: STAN result values for the different estimated variables in the temperature-based model, including the median and the 95% CI posterior distributions, rhat values and effective sample sizes.

| Variable | Name | Q05 | Median | Q95 | *rhat* | ESS |
| --- | --- | --- | --- | --- | --- | --- |
| $\beta$ | General intercept | -0.019 | 0.204 | 0.445 | 1.0040 | 1715 |
| $\beta_{F} \sigma$ | Between-fish standard deviation | 0.360 | 0.432 | 0.531 | 1.0007 | 3657 |
| $\beta_{R} \sigma$ | Between-replicates standard deviation | 0.137 | 0.181 | 0.245 | 1.0013 | 1295 |
| $\beta_{L^{2}}$ | Slope for size effect | -0.014 | 0.098 | 0.210 | 1.0016 | 1968 |
| $\beta_{T}$ | Slope for temperature effect | -0.170 | -0.104 | -0.043 | 1.0004 | 2762 |
| $\beta_{T^{2}}$ | Slope for temperature^2^ effect | -0.056 | 0.043 | 0.149 | 1.0000 | 2929 |
| $\beta_{Diet 60\%}$ | Intercept for 60% diet level | 0 | 0 | 0 | - | - |
| $\beta_{Diet 75\%}$ | Intercept for 75% diet level | -0.324 | -0.022 | 0.254 | 1.0016 | 1381 |
| $\beta_{Diet 90\%}$ | Intercept for 90% diet level | -0.420 | -0.120 | 0.169 | 1.0008 | 1712 |
| $\beta_{R}$ | Slope for ration size effect | -0.118 | -0.050 | 0.013 | 0.9999 | 2888 |
