# Supplementary material for: Size-independent, between-individual variability in feed ingestion rate in European seabass (Dicentrarchus labrax)
Source: PLoS One. 2026 Apr 16;21(4):e0347113. doi: 10.1371/journal.pone.0347113 (PMC13086339; doi:10.1371/journal.pone.0347113)
Supplement: S4 Table — The expected log predictive density (elpd_loo) provides a measure of model fit, with higher values indicating better predictive performance. The effective number of parameters (p_loo) reflects model complexity, and the LOO Information Criterion (looic) facilitates comparison across models (lower is better). Standard errors quantify the uncertainty in these estimates. (DOCX) [file pone.0347113.s008.docx]

Table S4: Summary statistics from approximate leave-one-out cross-validation (LOO) assessing the out-of-sample predictive accuracy of the fitted Bayesian models (stress-based: SB and temperature-based: TB). The expected log predictive density (elpd_loo) provides a measure of model fit, with higher values indicating better predictive performance. The effective number of parameters (p_loo) reflects model complexity, and the LOO Information Criterion (looic) facilitates comparison across models (lower is better). Standard errors quantify the uncertainty in these estimates.

| Metric | Model | Value | SE |
| --- | --- | --- | --- |
| elpd_loo | SB | -4333.5 | 1933.7 |
|  | TB | -4207.1 | 1873.6 |
| p_loo | SB | 2855.4 | 1925.5 |
|  | TB | 2729.9 | 1865.9 |
| looic | SB | 8667.0 | 3867.4 |
|  | TB | 8414.2 | 3747.2 |
